# Preimplantation Genetic Testing for Chromosomal Abnormalities: Aneuploidy, Mosaicism, and Structural Rearrangements

**DOI:** 10.3390/genes11060602

**Published:** 2020-05-29

**Authors:** Manuel Viotti

**Affiliations:** Zouves Foundation for Reproductive Medicine and Zouves Fertility Center, 1241 East Hillsdale Blvd, Suite 100, Foster City, CA 94404, USA; manuel@zouvesfoundation.org

**Keywords:** PGT-A, PGT-SR, mosaicism, embryo genetics, chromosomal abnormality

## Abstract

There is a high incidence of chromosomal abnormalities in early human embryos, whether they are generated by natural conception or by assisted reproductive technologies (ART). Cells with chromosomal copy number deviations or chromosome structural rearrangements can compromise the viability of embryos; much of the naturally low human fecundity as well as low success rates of ART can be ascribed to these cytogenetic defects. Chromosomal anomalies are also responsible for a large proportion of miscarriages and congenital disorders. There is therefore tremendous value in methods that identify embryos containing chromosomal abnormalities before intrauterine transfer to a patient being treated for infertility—the goal being the exclusion of affected embryos in order to improve clinical outcomes. This is the rationale behind preimplantation genetic testing for aneuploidy (PGT-A) and structural rearrangements (-SR). Contemporary methods are capable of much more than detecting whole chromosome abnormalities (e.g., monosomy/trisomy). Technical enhancements and increased resolution and sensitivity permit the identification of chromosomal mosaicism (embryos containing a mix of normal and abnormal cells), as well as the detection of sub-chromosomal abnormalities such as segmental deletions and duplications. Earlier approaches to screening for chromosomal abnormalities yielded a binary result of normal versus abnormal, but the new refinements in the system call for new categories, each with specific clinical outcomes and nuances for clinical management. This review intends to give an overview of PGT-A and -SR, emphasizing recent advances and areas of active development.

## 1. Introduction

The modern fertility clinic aspires to achieve a healthy birth using a single embryo transfer (SET). Historically, the transfer of two or more embryos simultaneously was a common ART industry practice, at times resulting in multiple pregnancies and their associated clinical complications [1,2]. A patient’s in vitro fertilization (IVF) treatment will frequently produce several embryos available for transfer, and the challenge becomes how best to rank a cohort of embryos in order of highest to lowest likelihood of success. The most rudimentary method to grade embryos is by their appearance, a practice that has been performed since the earliest days of IVF [3]. Although excellent standardization systems for an assessment of embryo morphology have been developed [4], the practice remains subjective, and morphology alone has been shown to be a limited predictor of implantation [5]. When it was understood that early human embryos often harbor chromosomal abnormalities, employing chromosomal profiling to deselect those with copy number and structural anomalies became an attractive prospect. The first application of chromosomal profiling in embryos was to determine their XX/XY status in patients with inherited X-linked conditions, by removing a representative cellular sample from each embryo and analyzing it with molecular methods [6]. This served as proof of concept that autosomal profiling could also become clinically applicable, setting the stage for the development of preimplantation genetic testing (PGT) for aneuploidy (-A) and structural rearrangements (-SR). Over the past two decades, the usage of PGT-A/-SR has experienced tremendous growth, and now accompanies a proportion of ART cycles in many parts of the world [7]. Vocal critics of the technology do exist, and as discussed below, not all clinical data from PGT-A have been positive. However, there are excellent arguments for the appropriate application of PGT-A when the limitations of the technology are understood.

**Figure 1:**
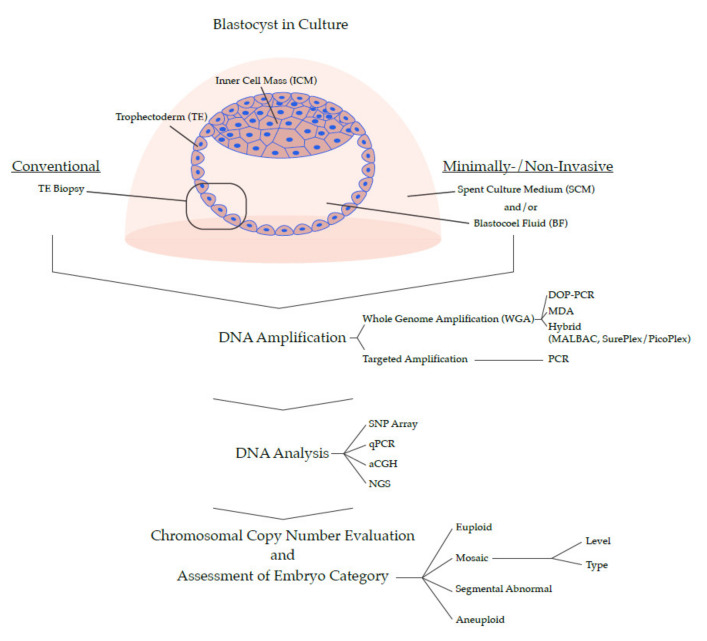
Overview of PGT Methods for 24-Chromosome Analysis.

At this point in time, PGT-A is experiencing a massive shift in how it categorizes embryos by their chromosomal profiles (Figure 1). A simple binary grouping of ‘normal’ or ‘abnormal’ has become insufficient. Recent data, which will be presented here, argues for a much more refined categorization, and encompasses embryos that are ‘euploid’, ‘aneuploid’ (whole chromosome, e.g., monosomy/trisomy), ‘mosaic’, and ‘segmental abnormal’. Additionally, the mosaic and segmental abnormal groups can be refined further according to their characteristics. The goal of such stratification is to obtain an enhanced ranking system to permit selection of the embryo with best likelihood of a positive clinical outcome.

From a technological standpoint, PGT-A is now coming to a watershed moment in its development and will be shaped by two seemingly opposing forces: simplicity and complexity (Figure 1). On one hand, there is a push to make the process easier, by developing a non-invasive version that would considerably simplify the sample collection step in the laboratory and make it available to a greater number of fertility clinics (but importantly, at the potential expense of data quality). On the other hand, there is demand for more complexity in the data by increasing the resolution of the genome, combining multiple genetic analyses (e.g., copy number and B-allele frequencies, or chromosomal and single gene profiling), or eventually sequencing the entire genome of a candidate embryo. Time will tell which path the technology will take, or whether several parallel versions of PGT-A will co-exist; what is certain is that the ‘genetic revolution’ has already transformed embryo selection in IVF and will continue doing so for years to come.

## 2. Human Reproduction and Chromosomal Abnormalities

The extent to which chromosomal abnormality affects human reproduction is noteworthy. Natural fertility in humans follows an inverse U-curve during maternal reproductive years, and evidence shows that embryonic chromosomal abnormality originating from meiotic errors during oocyte formation is the main cause for the reduced potential toward both ends of the curve [8]. This phenomenon is specific to humans (e.g., chimpanzees’ fertility potential remains uniform across maternal reproductive lifespan [9]), and is presumably linked to selective forces balancing risks and evolutionary fitness associated with human childbearing [8]. Even at the peak of a woman’s fertility, the incidence of chromosomal abnormality is not negligible—on average affecting ~20% of oocytes [8]. As a result, roughly half of all human preimplantation embryos harbor chromosomal abnormalities [10,11,12], when in comparison only 1% of early mouse embryos are chromosomally abnormal [13].

Together, these observations suggest that some degree of error-proneness during chromosome segregation in gametogenesis is beneficial to our species. Embryonic chromosomal abnormality should therefore not be regarded as an aberration, but rather an integral and programmed component in the natural process of human reproduction. Further accentuating this point, there is no difference in rates of implantation, miscarriage, and live births between advanced maternal age (AMA) patients and non-AMA patients when chromosomally normal embryos are used for intrauterine transfer [14,15], meaning that the age-related decline in fertility is solely controlled by embryonic abnormality. For the sub-fertility and infertility patient attempting treatment through ART, this peculiarity of human reproduction poses significant problems. The transfer of chromosomally anomalous embryos in the IVF clinic results in failed implantation, miscarriage, or congenital conditions.

## 3. Overview of Chromosomal Abnormalities in Embryos: Types, Mechanisms, Incidence, and Medical Implications

### 3.1. Aneuploidy

Aneuploidy is the most common genetic abnormality found in humans, and its high incidence in embryos is the main cause for failed implantation, pregnancy loss, and congenital birth defects [16]. Diploid cells normally contain 46 chromosomes, a state known as euploidy. Aneuploidy is an altered condition involving a deviation in copy number from multiples of 23. Typical examples are monosomy or trisomy, respectively resulting in 45 or 47 chromosomes. Aneuploidy can affect numerous chromosomes in a cell, sometimes referred to as complex aneuploids, or result in nullisomy or polysomy, where none or multiple copies of an individual chromosomes are present.

Aneuploidy in preimplantation embryos is primarily a result of chromosomal/chromatid segregation errors occurring at meiosis (in sperm or egg), uniformly affecting all cells in resulting embryos. Those mechanisms can be broadly grouped into (1) Non-disjunction errors (where homologous chromosomes or sister chromatids fail to separate) and (2) Premature separation (where homologous chromosomes or sister chromatids separate early). A correctly functioning cohesion apparatus between paired entities is therefore vital to preserve euploidy. The vast majority of meiotic errors occur in maternal meiosis (90%–99%) [16,17], of which recent studies estimate ~50%–70% originate at meiosis I, and ~30%–50% at meiosis II [17,18,19,20]. Aneuploidy is far less likely to derive from meiotic events in the father, with estimates ranging between 1%–10% [17,21,22]. The dissimilarity in percent aneuploidies originating from female and male gametogenesis is ascribed to several differences which make meiosis in the oocyte more error-prone, including: (1) Oocyte crossover structures during recombination are more frail, (2) Subdued prophase checkpoint control, (3) Decreased efficiency of the spindle assembly checkpoint (SAC), (4) Lower requirements for chromosomal alignment in the spindle equator for onset of anaphase, and (5) Different cell cycle progression [16].

A large percentage of spontaneous abortions in natural and ART pregnancies are ascribed to aneuploidies, as highlighted by a recent study cytogenetically analyzing 2564 samples of fetal tissue from first trimester miscarriages, detecting abnormal karyotypes in 49.5% of cases [23]. Furthermore, about ~4% of stillbirths and ~0.3% of newborns harbor aneuploidies [24]. Because of the strong in utero selection against aneuploidy, its ‘true’ incidence is determined from studies in fertilized eggs, which is currently impossible for natural conceptions [16]. Overall, about half of ART-generated preimplantation embryos contain uniform aneuploidy when assessed by PGT-A [11,12,25], but there is a distinctive maternal age-related effect. Three studies, each analyzing over 12,000 human blastocysts from ART patients using different genetic testing platforms, came to comparable conclusions: average percent euploid embryos increased from ~60% to ~75% between maternal ages 22 and 28, dipping to ~60% by age 35, followed by a steady decline to ~40% by age 40 until reaching ~10% by age 45 [26,27,28]. Logically, the incidence of aneuploidy followed the inverse trend, exhibiting its lowest incidence of ~25% at ages 28–29, and a steep rise after age 35, reaching its peak of 90% at age 45 [27]. These observations were recently replicated by a large, single-reference laboratory analysis of over 100,000 blastocyst-stage embryos [11]. Mechanistically, this age-related increase in aneuploidy is thought to be an effect of the prolonged prophase I arrest in oocytes (a state that can last up to 50 years), which results in gradual degradation of the meiotic apparatus [29]. In contrast, the incidence of aneuploidy does not correlate with paternal age [30,31].

Embryonic aneuploidy is also believed to be affected by environmental factors. Some lifestyle conditions of parents, such as obesity, smoking, exposure to radiation, and use of contraceptives have been proposed as candidates to increase meiotic errors [30]. Another source is the hormonal stimulation protocol [32,33]. There is documented variability between IVF clinics in the percentage of aneuploid embryos that are generated [25], suggesting that protocols of oocyte retrieval, intracytoplasmic sperm injection (ICSI), and culture conditions might also contribute to the incidence of meiotic errors.

Aneuploidies analyzed at the blastocyst stage are not evenly distributed over the 23 sets of chromosomes. A review of 5,000 embryos showed that aneuploidies in chromosomes 15, 16, 21, and 22 (relatively small chromosomes) were the most common, while those in chromosomes 1 to 6 (the largest autosomes) were the least common, although by and large the incidence of monosomies and trisomies at this developmental stage were quite similar [30]. A clear exception was chromosome 9, for which trisomies far offset monosomies [30], possibly indicating the presence of genes with essential dosage effects for early development.

All autosomal monosomies and most autosomal trisomies are embryonic lethal, the exceptions being trisomies 13, 18, and 21 which occasionally lead to births resulting in Patau, Edwards, and Down’s Syndromes, respectively. The fact that Down’s patients can survive well into adulthood suggests that merely having an abnormal chromosomal copy number might not affect viability per se, and rather, it is abnormal transcriptional dosage of genes located in the affected chromosome that compromises development [34]. Incidentally, chromosome 21 is comparatively small and contains relatively few genes, possibly explaining the long-term survival of cells harboring the abnormality. Aneuploidies affecting the sex chromosomes can have a range of clinical implications from undetectable to severe or lethal, depending on the copy number and chromosome affected; 47,XXX and 47,XYY typically result in phenotypically normal females and males, 45,X and 47,XXY lead to Turner and Klinefelter Syndromes, but the X chromosome is absolutely essential as its absence invariably leads to embryonic demise. The absence of one entire set of chromosomes (haploidy) in embryos, or the presence of extra sets (polyploidy), is incompatible with human life. However, it must be noted that these conditions do not fall under the strict definition of aneuploidy (as their chromosomal counts result in multiples of 23).

### 3.2. Chromosomal Mosaicism

The definition of chromosomal mosaicism is the co-presence of cells with two (or more) different chromosomal constitutions. In the context of PGT-A, the most relevant type is the mix of euploid and aneuploid cells (sometimes referred to as diploid-aneuploid mosaics, hereafter simply referred to as mosaic) because in recent years such embryos have been shown to result in healthy pregnancies when transferred in the clinic [35,36,37,38,39,40].

Mosaicism originates from mitotic events during post-zygotic development. The best characterized types of mitotic errors resulting in mosaicism are sister chromatid malsegregations [12]: anaphase lagging, mainly resulting in one normal and one monosomic daughter cell (although other patterns of chromosomal inheritance are possible [41]), and non-disjunction, leading to reciprocal trisomic and monosomic daughter cells. Other types of mitotic error resulting in mosaicism are endoreplication, (a diploid cell becomes trisomic by excessive replication of a chromosome), formation of micronuclei (the aberrant establishment of independent nuclear membrane-encapsulated chromosomal material), and centriole/centrosome dysregulation affecting chromatid segregation [42].

The observation that monosomies are commonly found without reciprocal trisomies in mosaic embryos indicates that anaphase lagging might be more frequent than non-disjunction during mitotic errors [43,44]. All these events are mainly attributed to three factors associated with preimplantation embryos: Relaxed control of the cell cycle, aberrations of the centrosome and mitotic spindle, and defects in chromosome cohesion [45]. Cell intrinsic regulation, correction mechanisms, and cell cycle checkpoints are subdued during the first days of post-zygotic development, which is characterized by rapid expansion governed by a strained system of maternal factors before activation of the embryonic genome [45,46]. In this regard, early embryos are comparable to cancers, which experience dysregulated cell cycle control and high rates of aneuploidy [34].

Embryonic mosaicism was first described in 1993 by Delhanty and colleagues performing fluorescent in situ hybridization (FISH) in cleavage-stage embryos, observing different chromosomal counts between cells of a single conceptus [47]. The same phenomenon was also described later in blastocysts by Evsikov and Verlinsky [48]. Modern PGT-A methods can detect mixes of euploid and aneuploid cells with high accuracy, but there is variability between clinics regarding the reported incidence of embryos classified as mosaic with PGT-A at the blastocyst stage. Most recent estimates range between 4%–22% [38,40,49,50,51]. The reasons for this variation are biological (conditions in laboratories affecting rates of mosaicism) [46,51], as well as technical (lack of standardized system to interpret and report PGT-A results); discussed further below. Estimating rates of mosaicism in preimplantation embryos from natural conceptions is challenging, but observations made in bovine cleavage-stage embryos have revealed that in vitro production engenders a significantly higher incidence of mosaicism than in vivo conception [52]. However, karyotype analysis of neonate umbilical cord blood and placenta showed no difference in rate of mosaicism between samples of natural and IVF conceptions in humans [53].

Contrary to meiotic errors and their age-related accumulation, there appears to be a uniform baseline risk for mitotic errors throughout the maternal reproductive years [54]. Nonetheless, there is a demonstrated reduction in incidence of mosaicism between cleavage and blastocyst stages [54], which further declines through gestation. Mosaicism is detected in ~2.1% of chorionic villus samples (CVS), which tests placental cells [23], and < 0.2% of newborns are estimated to be mosaic, although this is difficult to assess since routine karyotyping of newborns is not performed [49,55]. The attrition of mosaicism through development indicates the presence of selective forces that either cause mosaic embryos to perish, or elicit preferential demise of aneuploid cells in mosaic concepti.

Whether a mitotic error occurs early in development or later influences the percent of cells in the conceptus that contain the abnormality; the closer to the zygote stage, the higher the number of cell divisions that can propagate the aneuploidy. The load of abnormal cells is likely to influence the survival of embryos. Bolton and colleagues have used murine chimera experiments to model mosaicism, showing that embryos with high aneuploid load invariably died [56]. However, embryos with low aneuploid load survived and eliminated abnormal cells by apoptosis in the inner cell mass (ICM, the precursor to the fetus) or attenuated proliferation in the trophectoderm (TE, the precursor to the placenta). This is in accordance with the well-documented fact that aneuploidy generally hinders rates of cell proliferation [34]. A recent study by Mashiko and colleagues used live imaging on an engineered mouse strain with fluorescent nuclei and captured, for the first time, spontaneous mitotic errors and the establishment of mosaicism in developing preimplantation embryos [57]. They observed a correlation between the severity of aneuploidy in the abnormal cell compartment and likelihood to arrest before reaching the blastocyst stage. When transferred, murine blastocysts with visually-confirmed mosaicism resulted in births of healthy pups, suggesting a negative selection against the aneuploid cells through gestation [57]. Extended in vitro culture experiments by Popovic and colleagues with 18 mosaic human blastocysts showed that 58% remained viable in Day 12 outgrowths, of which 80% had resolved the mosaicism and presented euploid profiles, while 20% retained the mosaicism [58]. A mechanism of targeted apoptosis and/or proliferative out-competition of aneuploid cells by euploid cells could explain how a mosaic human embryo might become a healthy baby, as aneuploid cells are effectively diluted out in the course of development. In point of fact, experiments using immunofluorescence have shown significantly different patterns of mitosis and cell death between euploid and mosaic embryos [40], presumably reflecting a process in mosaic embryos of purging aneuploid cells by their directed demise and compensatory proliferation of euploid cells.

An alternative mechanism for the generation of mosaic embryos has been proposed, in which a meiotic error in germ cells could be followed by a mitotic event post-zygotically, ‘correcting’ the initial aneuploidy. Processes of such intra-cellar correction have been proposed, in which trisomic cells lose the extra chromosome (trisomic rescue) or monosomic cells duplicate the singular chromosome to restore disomy (endoreplication). To date, while one study reported evidence of trisomic rescue pre-zygotically (in oocytes, where a meiosis I error was corrected at meiosis II) [59], such mechanisms have not been convincingly demonstrated in human embryos. These hypothetical events would often result in uniparental disomy (UPD), which in IVF embryos is extremely rare, estimated at 0.06% [60]. Experimental data from extended in vitro culture experiments suggest the contrary, that uniform (non-mosaic) euploid or aneuploid embryos tend to maintain their initial ploidy in both ICM and TE lineages [58]. An embryo with aneuploidy stemming from a meiotic event is therefore bound to retain the chromosomal abnormality and result in failed implantation, miscarriage, or liveborn syndrome.

The medical consequence of mosaicism is a subject of current investigation. Regarding clinical outcomes, recent data indicate that mosaic preimplantation embryos can result in successful pregnancies and healthy births, but at lower rates compared to euploid embryos [35,36,37,38,39,40]. In gestation, mosaicism is implicated in confined placental mosaicism (CPM), where cells with different ploidy are present specifically in the placenta and fetal cells are normal. The majority of pregnancies diagnosed with CPM continue to term with no complications [61], but some result in intrauterine growth retardation (IUGR), placental insufficiency, and potentially miscarriage [62,63]. The real medical meaning of mosaicism in newborns and adults remains elusive. On one hand, chromosomal mosaicism has been associated with various conditions, including psychiatric disorders, autoimmune disease, and congenital malformations [64]. On the other hand, there is a documented high incidence of mosaicism in various tissues of healthy persons including brain, blood, and skin [65,66].

### 3.3. Segmental *Abnormalities*

Segmental abnormalities (sometimes referred to as partial aneuploidies or structural aberrations) affect sub-chromosomal sections, and in the context of PGT-A they typically denote regional losses or gains. The size of a segmental abnormality detectable by modern PGT-A platforms is usually 10–20 Mb and above [67,68], but in some instances the identified segments are as small as 1.8 Mb [69].

Segmental abnormalities originate from faulty corrections of chromosome breakage, so their etiology is altogether different to that of whole chromosome aneuploidies. Double strand breaks (DSBs) occur during DNA synthesis when replicative forks stall and collapse, for reasons such as DNA damage, absence of DNA-synthesis constituents, or strain associated with DNA secondary structure [70,71]. In gametes, there are programmed strand breaks to enable meiotic recombination, which can go awry and result in chromosomal breakage [72]. DSBs can also result from exogenous factors such as oxidative stress or the effect of mutagens. Generally, DSBs elicit a DNA repair mechanism, and failure to execute it typically activates the apoptotic process [73]. Those pathways are often compromised in preimplantation embryos, which are characterized by rapid cell division, compromised repair mechanisms, lax cell cycle checkpoints, and deregulated apoptosis [74]. When a cell ‘repairs’ a DSB incorrectly, it can result in duplication or deletion of the segment containing the break.

Segmental copy number variants can originate pre- or post-zygotically, respectively affecting all embryonic cells or only a subset. An estimated ~32% of segmental abnormality is meiotic in nature and present throughout the cells of the remaining blastocyst [75], meaning that the majority of instances are of mitotic origin and present in a mosaic pattern [75,76]. Segmental abnormalities can occur in any chromosome, their incidence across the genome largely correlates with chromosomal size, and the frequency of losses and gains is roughly equal [75]. One possible outlier is the high incidence of segmental gains in the q-arm of chromosome (Chr) 9 [74,75]. A study has described loci in the genome of preimplantation embryos with higher likelihood of segmental abnormalities, possibly related to heterochromatic composition of those regions [70].

Approximately ~6%–15% of ART-created blastocysts contain segmental abnormalities when evaluated by current PGT-A methods with the described resolution, either exclusively or in conjunction with whole chromosome aneuploidies [70,74,75]. When only considering instances with no concomitant whole chromosome aneuploidy, the incidence of blastocysts with segmental losses or gains is ~2.4%–7.5% [11,70,74,75]. Incidence of segmental abnormalities in blastocysts do not correlate with medical indication or patient age [70,74,75]. Segmental abnormalities are thought to account for 6% of clinical miscarriages [77] and affect close to 0.05% of newborns [78]. In persisting to term, segmental copy number variants can result in various syndromes and conditions, for example Cri-du-chat (caused by terminal deletion in the p arm of Chr 5), or Charcot–Marie–Tooth disease type 1A (caused by an interstitial duplication in the p arm of Chr 12) [79].

### 3.4. Structural Rearrangements

Balanced translocations, Robertsonian translocations, insertions, and inversions are abnormalities that change the natural order of chromosomal segments, but leave copy numbers unaltered. Carriers of such anomalies are typically asymptomatic, but recombination and sorting at meiosis can produce chromosomal copy number abnormalities in egg and sperm. This results in fertility problems, increased likelihood of pregnancy loss, and heightened chances of producing offspring with physical and mental disabilities. Therefore, whereas PGT-A is a screening tool for chromosomal abnormalities that arise spontaneously, PGT-SR is a targeted test performed when known chromosomal abnormalities are present in parental genomes. PGT-SR requires a personalized review of parental karyotypes, as the resulting cohort of embryos are tested for instances of recombination producing unbalanced chromosomal configurations in at-risk regions. Occasionally, PGT-SR is also performed when a familial abnormality involves segmental copy number variants, typically with small deletions and duplications that result in relatively mild symptoms in prospective parents. Individualized case evaluation determines whether a given PGT platform has the resolution to detect copy number alterations for a specific segment.

Most PGT-SR is performed with standard PGT-A platforms (as long as the affected regions are above the platform’s resolution) as embryos with unbalanced translocations can be identified by displaying segmental losses or gains in the regions involved in the translocation. Nonetheless, this type of PGT-SR cannot distinguish between those embryos in a cohort that are euploid and those that carry the balanced translocation. Although replacing balanced embryos should result in phenotypically normal births, the offspring will, later in life, encounter the same problems as their carrier parents, including reduced fertility, increased miscarriages, and having affected children. A more sophisticated version of PGT-SR can differentiate between euploid and balanced embryos, by analyzing the sequences or genetic markers in/around breakpoint regions [80,81,82,83]. Although much more involved than routine PGT-SR, this method can be opted for when patients want to preclude the transfer of balanced embryos, or when the reciprocal translocation affects the X chromosome, considering that the phenotype of balanced carriers is unpredictable due to random inactivation of one X chromosome in female embryos [84].

## 4. Development of PGT for Chromosomal Abnormalities

### 4.1. Early Methods of PGT-A

The first clinical instance of chromosomal profiling in human embryos occurred in 1990, but not for aneuploidy [6]. Handyside and colleagues analyzed individual blastomeres of cleavage-stage embryos (Day 3 post fertilization) from a mother that carried a X-linked condition, and amplified a Y-specific repeat sequence using polymerase chain reaction (PCR). In 1992, fluorescent in situ hybridization (FISH) using X- and Y- probes were used in the clinic, again to treat families at risk of transmitting sex-linked disorders [85]. The first direct investigation of embryonic aneuploidy occurred in 1993, using a FISH test for chromosomes with known liveborn syndromes (13, 18, and 21) in addition to the sex chromosomes (X, Y) [47,86,87]. This was the birth moment of PGT-A and PGT-SR. FISH can be adapted to examine several regions within a single chromosome by using multiple probes, for example, by simultaneously using one centromeric and two sub-telomeric probes. This is applicable for patients carrying balanced translocations, identifying the embryos that inherited the translocation in an unbalanced way.

Over the two decades that followed, cleavage-stage PGT-A by FISH grew as an adjunct to the IVF cycle under the recommendations to transfer normal embryos and discard abnormal embryos. Nonetheless, the technology remained limited by the number of chromosomes each test could examine. Although some groups have been successful at implementing this technology clinically to examine up to 12 chromosomes at once [88], this still left half of the chromosomes undiagnosed. This important limitation, as well as the demanding nature of the technique, has led to FISH being largely replaced with other PGT-A platforms. FISH still plays a role in some specific PGT-SR cases, for example, in instances of very distal breakpoints, such that conventional PCR-based methods are inadequate [89]. Furthermore, blastomere collection at the cleavage stage has the potential to decrease embryo viability [90], encouraging development of biopsy isolation techniques at other embryonic timepoints.

Another potential source of genetic material to be evaluated both in its effect on embryo viability and its ability to reflect the chromosomal constitution of the resulting embryo was that of polar bodies (PBs) [91]. Most aneuploidies originate from meiotic errors in the female, such that the PB can contain the reciprocal aneuploidy to that of the associated oocyte (or zygote). For example, in a non-disjunction event, a PB could contain monosomy 21 while the oocyte contains trisomy 21. Nonetheless, many types of aneuploidy in the embryo would not be detected by this method (non-reciprocal aneuploidies, paternal meiotic errors, mitotic events). Furthermore, given that 30%–50% of oocyte-derived chromosomal imbalances originate at the second meiotic division [19,20], there is a necessity to analyze both PBs, considerably increasing the cost of the procedure. In addition, a number of studies investigating PGT-A based on PB analysis have shown high rates of false results [19,92], and negative impact on embryo viability [93]. Irrespective of these caveats, PB analysis persists in countries that legally restrict direct embryo testing [7].

Together, these shortcomings of the original, FISH-based version of the technology (sometimes referred to as PGS 1.0), combined with clinical analyses showing poor performance [94], set the stage for the development of 24-chromosome analysis.

### 4.2. Development of PGT-A for All Chromosomes

Expansion of the system to investigate all chromosomes (sometimes referred to as PGT-A 2.0) was transformative to the field. A diploid human cell contains approximately 6.6 picograms of DNA, which is too little (even when 5–10 cells are collected in a biopsy) to perform molecular analysis by conventional approaches. The key step was the development of amplification methods that multiplied the DNA in the biopsy. This process has been dubbed whole genome amplification (WGA), and generates sufficient DNA with appropriate genome coverage for full 24 chromosome analysis. Several chemistries have been developed or adapted for this purpose, each with its strengths and weaknesses: Degenerate oligonucleotide primed PCR (DOP-PCR), multiple displacement amplification (MDA), and hybrid methods, including multiple annealing and looping based amplification cycles (MALBAC), and SurePlex/PicoPlex, all comprehensively reviewed and compared before [95,96]. Once the DNA is amplified, it can be used for varying methods of downstream analysis: comparative genome hybridization (CGH) [97,98], arrayed CGH (aCGH) [99,100], single nucleotide polymorphism (SNP) arrays [101], or next generation sequencing (NGS) [67,102]. Ultimately, their goal is one and the same; revealing aneuploidy by identifying regions of the genome that are under- or overrepresented. Another strategy, known as targeted amplification, involves PCR-based replication of strategic regions spread out across the genome. The products can subsequently be quantified by quantitative PCR (qPCR), or by sequencing [103,104]. In comparison to WGA methods of PGT-A, targeted amplification can increase sequencing depth in the targeted regions, but might do so at the expense of breadth of genomic coverage.

At present, the optimal PGT-A solution lies at the intersection between sequence information (coverage breadth and depth) and cost/ease of use. Among all the options, WGA-based NGS has emerged as the most popular method across the PGT-A landscape. The technique has low depth of sequencing, but relatively high breadth of genome coverage, which is suitable for copy number analysis. Decreasing cost of sequencing might make true, ‘deep’ whole genome sequencing for each embryo a reality in the future.

### 4.3. Contemporary PGT-A

In recent years, there has been a progressive, worldwide move toward biopsy collection at the blastocyst stage [7], where cells are removed from the TE (the precursor to the placenta). Isolation of a TE biopsy was first described in 1990 by Dokras and colleagues [105]. Since then, different approaches have been described and are continuously being evaluated and refined, for example the use of assisted hatching or laser manipulation [5,106,107,108,109,110,111]. Two advances in embryology have made the shift to the blastocyst stage possible. One was the development of more physiological culture media, making it possible to consistently grow embryos to the blastocyst stage [112,113]. The other was an improved cryopreservation method of rapid freezing (known as vitrification), which ensures very high survival of blastocysts [114]. Together, they have made it possible to grow embryos until days 5, 6 or 7 post fertilization, at which point TE biopsies are collected and blastocysts are vitrified and stored until PGT-A results are available and transfer is scheduled.

Collecting a blastocyst-stage TE biopsy is advantageous compared to a single blastomere (or two) at the cleavage stage, for several reasons, including: (1) A TE biopsy removes a smaller percentage of the total cells of the embryo, and only detaches cells destined to become placenta as opposed to fetal tissue, (2) Analyzing the content of 5–10 cells provides more DNA, decreasing the chances of failed reactions and technical artifacts due amplification bias or allele drop out (ADO) [115,116], (3) Assessing a group of cells of the TE makes it possible to detect mosaicism within the biopsy, (4) The documented attrition of inviable embryos between cleavage and blastocyst stage [15] translates into fewer unnecessary tests, and (5) The particularly high incidence of mosaicism at the cleavage stage [97], which becomes partly resolved by the blastocyst stage [117], reduces the likelihood of false results.

If performed properly by trained hands, surgically removing 5–10 TE cells is not thought to affect blastocyst viability in an appreciable manner. Perhaps the best evidence comes from an elegant double embryo transfer experiment with blastocyst pairs of similar grade, in which only one blastocyst underwent biopsy collection, showed equal likelihood of implantation [90]. Recently it was reported that even repeat biopsies with two subsequent freeze-thaw cycles did not significantly affect embryo viability [118]. Conceptually those findings are not entirely surprising, considering that monozygotic twinning arises from a split of an embryo at the blastocyst stage, meaning that half of the cells in the blastocyst (TE and ICM) are sufficient to sustain normal fetal development.

The PGT-A technology that is currently most widespread is WGA, followed by NGS [67]. Compared to aCGH, its popular predecessor, WGA-based NGS is thought to provide superior resolution and dynamic range [76,119,120], resulting in improved rates of favorable outcomes in the clinic [38,121,122,123]. Analysis at the blastocyst-stage involves pooling DNA from 5–10 cells in the TE biopsy. The bioinformatic analysis of resulting data produces a representative average karyotype profile encompassing all 24 chromosomes. A relatively low coverage sequencing (with ≤0.1× depth) suffices for robust chromosomal copy number analysis [102,124], explaining why prevalent NGS-based PGT-A methods are occasionally said to use ‘low-pass’ or ‘shallow’ sequencing. Reads are aligned to a human refence genome and grouped together into ‘bins’ spread out over the chromosomes. The karyotype profiles indicate the copy number for each bin, which can be uniform along a chromosome or variable, indicating segmental abnormalities. Clear nullisomies, monosomies, disomies, trisomies, tetrasomies, and so forth produce data aligning to respective copy numbers 0,1,2,3,4 etc. Profiles with values falling between whole numbers are consistent with intra-blastocyst mosaicism. For example, values aligning at 2.5 for a chromosome are consistent with a 50% mix of cells with disomy and trisomy in the TE biopsy. Currently, many laboratories utilize the 20%–80% span in karyotype profiles to define a region consistent with mosaicism [125]. When values deviate <20% from whole numbers, they are not considered in the mosaic region because they fall inside the normal noise range. Numerous groups have performed detailed mixing experiments of DNA or cells with different karyotypes to model mosaicism, generally finding that WGA-based NGS is excellent at identifying it in whole chromosomes as well as segmental regions [37,39,40,76,126,127].

It must be noted that technical noise, artifacts of imperfect WGA or sequencing reactions, and suboptimal biopsy collection techniques, might also result in profiles that falsely indicate mosaicism [116]. When results appear noisy the confidence of identifying true intra-blastocyst mosaicism is compromised. Currently there are no guidelines for how to deal with such cases. In addition, a TE biopsy containing cells with reciprocal aneuploidies (due to sister chromatid non-disjunction) might yield skewed results, including possibly a disomic looking karyotype and concealing an instance of mosaicism [128]. The probability of such an event is currently unknown but likely to be small, particularly considering that reciprocal aneuploidies are not the predominant pattern of mosaicism in embryos [43,44].

Most modern PGT-A platforms are validated to detect segmental copy number variants of 10–20 Mb or larger, but often have the capability to detect smaller segment variants, some as small as 1.8 Mb [69,76,129]. The resolution is crucial for detection of segmental losses or gains associated with characterized newborn syndromes, of which the majority are in the 1–10 Mb range [130]. Some are even smaller, warranting future efforts to further increase the resolution of routine PGT-A (see Section 7.2).

## 5. Considerations on the Clinical Use of PGT for Chromosomal Abnormalities

### 5.1. How Reliable Is PGT-A?

There is broad consensus that contemporary PGT-A methods are superbly robust at evaluating the content of the input material. Consistency in results can be confirmed by re-sequencing WGA material from TE biopsies, or re-amplifying and sequencing aliquots of DNA or cells from lines with known karyotypes. This type of exercise typically indicates near 100% technical accuracy [67,76,100,101,102,103]. However, there are potential biological sources of error in PGT-A that must be taken into consideration. A human blastocyst is comprised of 64–128 cells (equaling 6–7 post-fertilization cell divisions) [131], meaning that a 5–10 cell TE biopsy contains 3.9% to 15.6% of all cells in the conceptus. Due to chromosomal mosaicism, a concern is that the TE biopsy is an imperfect representative of the embryo [55]. Thus, even when PGT-A accurately reflects the content of the TE biopsy, the result is meaningless if the TE biopsy is a poor proxy for the associated blastocyst.

Estimating the incidence of false results due to embryo-scale mosaicism has been the goal of several studies–even in the era of FISH and aCGH (comprehensively summarized by Capalbo and Rienzi [132]), broadly showing high concordance rates between TE and ICM. A number of recent publications have conducted serial biopsy experiments in blastocysts donated to research using NGS-based PGT-A. Evaluation of concordance between samples can be computed in two ways: Ploidy concordance (compares overall status of euploidy/mosaicism/aneuploidy) or full karyotype concordance (compares chromosomal profiles to evaluate if they are identical). For example, two biopsies can be aneuploid (ploidy concordant) but contain different aneuploidies (full karyotype discordant).

Huang and colleagues analyzed ICM and four TE biopsies in 44 blastocysts originally categorized as whole chromosome aneuploid (e.g., monosomy/trisomy), observing all were aneuploid in the four biopsies (100% ploidy concordance) [133]. There was perfect karyotype concordance amongst the four biopsies in 39 blastocysts (88.6% full karyotype concordance). In 7 blastocysts with original categorization of segmental abnormality, ploidy concordance was 100% but full karyotype concordance was reduced to 57.4% [133]. A study by Sachdev and colleagues investigated 32 blastocysts and calculated per chromosome concordances between TE biopsy and ICM biopsy, determining rates of 99.5% for euploid results, 97.3% for aneuploid results, but only 35.2% for mosaic results [134]. A study by Lawrenz and colleagues compared PGT-A results from three biopsies for each of the 84 embryos included in the study: one blastomere biopsy, one TE biopsy, and one ICM biopsy [135]. The ploidy concordance between the blastomere and ICM was 69.8% for aneuploidy and 90.3% for euploidy, but concordance was higher between TE and ICM, namely 92.5% for aneuploidy and 93.2% for euploidy. Discordance between TE and ICM largely stemmed from the detection of structural abnormalities in either of the lineages. Of the blastomere-ICM discordances, 84.2% had an aneuploid blastomere but a euploid ICM, and 78.6% of times the matching TE biopsy did reflect the euploidy of the ICM. Thus, the findings of the Lawrenz study showed that a TE biopsy is a superior representative of the ICM (which will become the fetus) to a cleavage-stage biopsy.

Of note, the Huang, Sachdev, and Lawrenz studies did not consider intra-biopsy mosaicism, so ploidy was euploid/aneuploid binary (in which case typically low mosaic profiles become categorized as euploid and high mosaic profiles become categorized as aneuploid). The next set of studies considered mosaicism within individual biopsies. A study by Victor and colleagues compared a TE biopsy to its paired ICM, and observed that in 90 out of 93 blastocysts, a TE biopsy containing whole chromosome aneuploidy corresponded to whole chromosome aneuploidy in the ICM (96.8% ploidy concordance) [136]. In one case the ICM was euploid, and in two cases the ICM was mosaic. However, when considering TE biopsies with exclusively segmental abnormalities, the same study observed ploidy concordance with the ICM in only 2 out of 7 cases [136]. In another study, the same group re-biopsied embryos with an initial mosaic profile in the TE, and observed that the mosaicism was only reflected in 3 out of 8 cases (out of which two contained the reciprocal error) (40).

Popovic and colleagues analyzed two sets of blastocysts [126]. The first set had an original TE biopsy PGT-A result, which when compared to the ICM showed high concordance for whole chromosome aneuploidy (10 out of 10 samples) and segmental abnormality (6 out of 6). However, when the initial TE biopsy was mosaic, concordance with the ICM was seen in only 1 out of 4 samples, as the other 3 had euploid ICMs. For segmental mosaic TE biopsies, the ICM was aneuploid in 1 out of 4 samples, and the other 3 had euploid ICMs. The second set of blastocysts in the study had not been subjected to PGT-A in the clinic, and serial biopsies (1 ICM and 4 TE) were collected for each. Out of 21 blastocysts with a euploid ICM, 14 had perfect ploidy concordance in all biopsies (66.7%), while the remaining 32.3% had ploidy discordance in at least one TE biopsy. When excluding abnormalities of mosaic or segmental nature, the ploidy concordance across all biopsies increased to 95.2% of blastocysts. Out of 10 blastocysts with uniform (non-mosaic) aneuploidy in the ICM, 80% were aneuploid across all TE biopsies. Finally, out of 3 blastocysts with a mosaic ICM, all three had at least one euploid TE biopsy.

Navratil and colleagues compared a TE biopsy to the entire embryo, observing euploidy concordance in 18 out of 19 blastocysts (94.7%), and whole chromosome aneuploidy concordance in 58 out of 62 blastocysts (93.5%) [137]. Segmental abnormalities showed significantly reduced concordance, as it was only detected in 14 out of 31 blastocysts (45.2%), and mosaic TE biopsies correctly predicted the equivalent mosaicism in the remaining blastocyst in 7 out of 26 instances (26.9%) [137].

Ou and colleagues focused entirely on segmental abnormalities and reported concordance between original TE biopsy and entire embryo in 43 out of 63 cases (68%) [138] When a similar experiment was conducted by Girardi and colleagues with multifocal biopsies, this time collecting four TE biopsies and an ICM biopsy for each blastocyst, it was observed that segmental losses or gains in any single TE biopsy were predictive of the ICM ~50% of times, but only ~32% of embryos contained the same segmental abnormality in all five biopsies [75]. In stark contrast, that same group found there to be discordance of whole chromosome aneuploidy in only 4 out of 390 (~1%) analyzed TE-ICM pairs [75], although it must be noted that the study did not consider intra-biopsy mosaicism.

Another potential source of biological error in PGT-A is the cell-cycle state of the probed cells. The worry revolves around S-phase, when DNA is replicated in a way that is not even across the genome and begins multifocally in tens of thousands of genomic regions called origin of replication. The resulting PGT-A profile of a single cell in S-phase could theoretically produce false positive profiles consistent with segmental abnormalities [139]. All cells in a TE biopsy would need to be synchronized and spontaneously engage the same origin of replication (something that is highly variable among cells [140]) to falsely mimic a uniform segmental gain, which is implausible. Nonetheless, including one or more cells in S-phase in the biopsy could conceptionally result in ‘noisy’ profiles [141], possibly consistent with mosaic profiles. However, a study by Ramos and colleagues on cell lines and embryos has shown that this effect is negligible with contemporary WGA methods for PGT-A [142].

The emerging theme from this set of combined findings is that a single TE biopsy detecting either uniform euploidy or whole chromosome aneuploidy is an excellent predictor of the state of the remaining embryo (likely reflecting a meiotic error), but segmental abnormalities and intra-biopsy mosaicism (mitotic error) have a vastly reduced predictive power.

### 5.2. Discussion on the Clinical Merits of PGT-A

After the development of FISH-based PGT-A, a collection of studies describing its use in the clinic propelled the adoption of the technology [143,144,145,146]. However, a number of randomized control trials (RCTs) published between 2004 and 2010 produced controversial results regarding its effect on clinical outcomes, and a comprehensive meta-analysis performed by Mastenbroek and colleagues in 2011 ultimately showed no benefit of PGT-A, and even described a detrimental effect [94]. This was true for all patient groups analyzed: good prognosis, advanced maternal age, and repeat implantation failure. The findings were mainly ascribed to four principal limitations of PGT-A at the time: (1) FISH only evaluated a subset of chromosomes, (2) Biopsy of one cell (or two) at the cleavage stage, a preimplantation timepoint with a particularly high incidence of mosaicism [146,147], potentially leading to false results, (3) Harm done to the embryo by removing a considerable portion of its biomass, (4) Technical challenges of producing clear results by FISH on a single cell. While a subsequent RCT by Rubio and colleagues showed a positive effect in AMA patients [148], the findings of the Mastenbroek meta-analysis in 2011 largely discredited FISH-based PGT-A [94]. However, almost concurrently to its publication, new technologies permitting 24-chromosome analysis in blastocysts were validated and launched for clinical use [98].

Since the advent of 24-chromosome PGT-A, an extensive body of evidence, encompassing numerous observational studies and RCTs, describes the benefits of the technology on clinical outcomes. Some of the most prominent are highlighted here.

The first RCT in the era of 24-chromosome analysis was published in 2012 by Yang and colleagues, where aCGH-based PGT-A and morphology assessment were used in one arm of the study (group A, *n* = 425 blastocysts), compared to morphology assessment alone in the other arm (group B, *n* = 389 blastocysts) [149]. Participants were all good-prognosis patients under 35 years old, and received fresh single embryo transfer (SET) at day 6. Group A had a better implantation rate than group B (70.9% and 45.8%, respectively; *p* = 0.017), as well as higher ongoing pregnancy rate (69.1% vs. 41.7%, respectively; *p* = 0.009).

That same year (2012), Scott and colleagues published a ‘non-selection’ study using a SNP array platform in which 255 cleavage- or blastocyst-stage embryos were evaluated by PGT-A, but results were only unblinded after clinical outcomes were known [150]. They observed a high predictive value for aneuploid results, with 96% of embryos designated as aneuploid failing to sustain implantation, while embryos classified as euploid resulted in sustained implantation 41% of times, which was significantly higher than the 28.2% overall rate of all embryos transferred.

In 2013, Scott and colleagues performed an RCT using qPCR and fresh embryo transfers, showing that the 134 blastocysts in the PGT-A group had a significantly higher sustained implantation rate compared to the 163 blastocysts in the control group (66.4% vs. 47.9%, respectively), as well as higher delivery rates from the group of embryos that implanted (84.7% vs 67.5%) [151].

Forman and colleagues published a noninferiority RCT in 2013, comparing the transfer of 89 single euploid blastocyst evaluated by qPCR compared with transfer of 86 pairs of unscreened blastocysts [152]. Ongoing pregnancy rates were comparable between the two arms (60.7% vs. 65.1%, respectively), but importantly the PGT-A arm did not experience any multiple pregnancies compared to the 53.4% incidence observed in the control arm, thereby demonstrating that an important complication of IVF could be eliminated without compromising success rates.

In 2015, two separate meta-analyses performed by the teams of Dahdouh [153] and Chen [154] evaluated the three blastocyst-stage RCTs detailed above and eight observational studies, clearly demonstrating the global positive impact of 24-chromosome PGT-A on clinical outcomes. Since then, a noteworthy large observational-cohort study in AMA patients published in 2019 by Sacchi and colleagues included the comparison of a blastocyst-stage qPCR PGT-A group (*n* = 201) with a blastocyst-stage group without PGT-A (*n* = 1,147), showing significantly increased live birth rate per transfer with PGT-A (40.3% vs. 23.4%, respectively) and lower miscarriage rate (3.6% vs. 23.4%, respectively) [155].

Furthermore, 24-chromosome PGT-A has also shown substantial benefits at non-blastocyst stages. An RCT published in 2017 on cleavage stage embryos conducted by Rubio and colleagues included 100 AMA patients in the aCGH PGT-A arm and 105 AMA patients in the control arm, and showed overall improved outcomes in the PGT-A group, including lower miscarriage rates (2.7% vs. 39% for control) and increased rate of delivery after first transfer attempt per patient (36.0% vs. 21.9%), significantly decreasing the number of transfers needed and time to pregnancy [156]. In a randomized clinical trial from 2018, Verpoest and colleagues showed benefits of aCGH PGT-A of PBs [157]. In total, 249 embryos with associated PB evaluation resulted in 50 live births (20.1%), compared to 440 of embryos with no PB evaluation resulting in 45 live births (10.2%). These findings are particularly important for countries that forbid culturing embryos to the blastocyst stage [7].

It has become evident that the transfer of a chromosomally normal embryo does not guarantee a favorable outcome, as other factors (e.g. maternal, environmental) influence its fate. Still, by 2019 the preponderance of data from 24-chromosome PGT-A had shown that excluding aneuploid embryos from transfer improved rates of positive outcome. The most recently published RCT used NGS technology [50]; however, it did not reveal any benefit to the overall patient population in the study. Those findings are discussed next.

### 5.3. A Note on the STAR Study

The much anticipated ‘Single Embryo Transfer of Euploid Embryo’ (STAR) study [50], a multicenter RCT, utilized a technical platform (NGS) and timepoint of biopsy collection (blastocyst stage) that, by current standards, should in principle exhibit the maximal benefit of PGT-A. Considering the overwhelmingly positive results from the previous 24-chromosome PGT-A RCTs (with perhaps less favorable conditions), the overall findings of the STAR study were unexpected: there was no general benefit in the euploidy plus morphology group compared to morphology assessment alone. Nevertheless, it must be emphasized that the STAR study focused on a singular, quite narrow question: Is PGT-A beneficial to good prognosis patients in the first, single embryo transfer?

In total, 34 clinics and 9 testing laboratories in the US, UK, Canada, and Australia contributed data from freeze-all cycles of patients aged 25–40 with at least two blastocysts available for transfer. IVF clinics operated independently, with no standardization regarding protocols of ovarian stimulation or embryo transfer. The PGT-A arm (euploidy plus morphology) comprised 330 patients, of which 274 ultimately received a transfer, while the control group (morphology only) comprised 331 patients, of which 313 ultimately received a transfer. Primary outcome of the study was ongoing pregnancy rate (OPR) at 20 week’s gestation. Regarding intention to treat (ITT), there was no significant difference in OPR between the two arms (41.8% vs. 43.5%, respectively; *p* = 0.65). Importantly, in the PGT-A arm, 42 patients had produced no euploid embryos and were automatically counted as producing no pregnancy. When analyzing results on a ‘per embryo transfer’ level, again the authors did not observe a statistical difference in OPR (50.0% vs. 45.7%, respectively; *p* = 0.32). However, post hoc analysis of patients in the 35–40 age group showed a statistically significant benefit of PGT-A on a per embryo transfer level (50.8% vs. 37.2%, respectively; *p* = 0.035).

It is nonetheless surprising that for this good prognosis patient group at first transfer no overall benefit was observed. Data show that a well-executed TE biopsy collection does not significantly affect blastocyst viability [90], but a poorly performed procedure will undoubtedly have a negative effect. Because of the multi-center nature of the STAR study, there was possibly variation in the expertise of TE biopsy procedure between clinics. Those with inexperienced technicians might have harmed embryos and reduced their viability, potentially annulling the benefits of PGT-A selection. To really assess this, a third study arm could have comprised biopsied embryos that are not tested, although it is evident that obtaining IRB approval and patient consent for that purpose would be problematic.

It must be clearly noted that the findings of the STAR study cannot be extrapolated to other patient groups. For example, no patients over 40 years old or with obvious clinical indications (RIF, RPL) were included in the study, which are the populations with highest incidence of embryonic aneuploidy [27], and therefore with most to gain from chromosomal evaluation. At least seven RCTs assessing PGT-A in different populations are currently registered at ClinicalTrials.gov, and will continue to provide valuable information on the matter.

### 5.4. Which Patients and Embryos Should Be Offered PGT-A?

While evidence from RCTs and observational studies for different patient categories continues to grow over time, PGT-A has historically been offered preferentially to patient groups believed to produce a high incidence of embryonic aneuploidy. For example, PGT-A follows the law of increasing returns with a gradually aging patient population because of the associated elevated incidence of aneuploidy [11,27]. Hence, the most common referral categories have been patients with indication of advanced maternal age (AMA, often defined as > 35 years), prior recurrent pregnancy loss (RPL, defined as 2 or more miscarriages prior to 20 weeks of gestation), prior recurrent implantation failures (RIF, commonly defined as three or more failures), and severe male factor (MF) infertility [11,158].

However, some fertility clinics elect to test every embryo [159]. The following two premises provide a rationale for offering PGT-A to all patients: (1) Whole chromosome aneuploidies in the TE are excellent predictors of aneuploidy in the ICM/remaining embryo (see Section 5.1), and (2) Aneuploid cells retain their ploidy status over time [58]. In fact, proposed intracellular self-corrective mechanisms, where aneuploid cell become euploid, have not been convincingly demonstrated in human embryos [45,46]. If the two above statements are correct, then a uniform (non-mosaic) aneuploid embryo identified by PGT-A will invariably retain its aneuploid condition, and fail implantation, miscarry, or result in congenital syndromes. Performing a large study with transfers of embryos classified as aneuploid by PGT-A is close to unimaginable at present, considering the need to obtain IRB approval and patient consent. Nonetheless, Munné and colleagues report ten instances of patients opting to transfer embryos with aneuploid results from 24-chromsome PGT-A, since no euploid or mosaic embryos were available to them [38]. The ten transfers resulted in one ongoing pregnancy resulting in an affected newborn that died at 6 weeks after birth. The lowest incidences of aneuploidy are observed in patients of ages 26–30, with the average percent of aneuploid embryos hovering around 25% [26,27]. This translates into a 1 in 4 chance of randomly selecting an aneuploid embryo for transfer if no PGT-A is performed, as there is only a moderate correlation between embryo morphology and ploidy [5,160]. Thus, even in the most favorable patient population with lowest incidence of aneuploidy, PGT-A can hypothetically have considerable impact. In spite of the STAR study results (discussed above), there is evidence from observational studies that PGT-A is beneficial in young, good-prognosis patients: It improved outcomes with young oocytes from egg donation cycles [161], and increased live birth rates per cycle in a statistically significant fashion in patients younger than 35 [162].

Needless to say, there are substantial costs associated with evaluation of chromosomal status in preimplantation embryos, most notably biopsy procedure, testing expenses, and genetic counseling. However, numerous studies have shown cost-effectiveness for various patient populations when compared to transfers without PGT-A [163,164,165]. The cost of performing the test is offset by savings from factors that accompany transfers of untested embryos, such as increased number of cycles, miscarriages, multiple gestations, and neonatal/ongoing aneuploidy-related conditions. Those factors also add non-tangible costs and the emotional toll to patients.

## 6. Refinement of PGT-A Categories

### 6.1. Management of Mosaic Embryos in the Clinic

In 2015, Greco and colleagues reported the clinical transfer of embryos classified as mosaic by PGT-A for the first time [36]. Eighteen consenting patients with no available euploid embryos opted for this treatment strategy, resulting in six healthy births. Up until that point, ‘mosaic’ embryos were largely grouped with uniform aneuploids in an ‘abnormal group’. Clinics and patients were understandably apprehensive of transferring embryos containing a proportion of aneuploid cells. Since then, several reports from different groups using NGS-based PGT-A (currently the most appropriate method to identify mosaicism) have described their experience of transferring mosaic embryos [35,36,37,38,39,40]. All studies coincided in one observation: Mosaic embryos had lower rates of implantation and higher likelihood of miscarriage than euploid embryos, but led to births with no overt medical conditions. This highly reproduced data in a combined > 800 transfers provide compelling evidence for mosaic embryos being considered for transfer as second priority after euploid embryos.

How can a mosaic blastocyst result in a healthy baby? What happens to the aneuploid cells? The described self-corrective mechanisms in mixed euploid and aneuploid cells (see Section 3.2) are likely responsible conversions of mosaic embryos into entirely euploid ones during development. In some instances, residual aneuploid cells might become diluted to the point of being medically negligible. In cases where aneuploid cells are confined to the TE lineage and persist through development, the potential consequence is CPM. Human placentas often contain islands of aneuploid cells, and are thought to be uniquely capable of adapting to chromosomal abnormalities, much more so than fetal tissues [166]. While most cases of CPM result in healthy babies [61], occasionally and depending on the aneuploidy involved and percent abnormal cells, the condition can result in miscarriage [167] (which, as noted, is more common after transfer of mosaic embryos than euploid embryos [123,168]). However, it must be noted that mosaicism observed in TE biopsies by PGT-A has not been observed in matching CVS or NIPT samples (which test placental DNA) in existing publications [39,40]. Together, these models provide a framework of how blastocysts with intra-biopsy mosaicism can result in healthy babies.

Another putative alternative to the fate of embryonic mosaicism is its persistence in fetal tissues through gestation, resulting in true fetal mosaicism (TFM) [169]. Some of the > 800 mosaic embryo transfers reported to date had matching amniocentesis information (which tests fetal DNA), from patients that opted to share results [36,39,40]. In the overwhelming majority of cases amniocentesis results were normal, and if an abnormality was detected it was independent of the mosaicism observed during PGT-A.

To date, there has been a singular instance of TE mosaicism matching the results of amniocentesis, reported by Kahraman and colleagues [170]. Interestingly, the authors observed a 35% mosaic loss of Chr 2 with PGT-A, and upon transfer, a reciprocal Chr 2 mosaic gain was detected at amniocentesis in 2% of cells. No pathological features were determined in detailed ultrasonography with normal fetal growth and no signs of IUGR. Birth of a healthy baby followed, in which peripheral blood chromosome analysis validated with fluorescence in situ hybridization showed 2% mosaic monosomy in Chr 2. Epithelial cells in a buccal smear were euploid. The patterns of reciprocal Chr 2 mosaicisms indicate a mitotic non-disjunction event early in embryogenesis, before or during segregation of embryonic (fetal) and extraembryonic (uterine) lineages. Hence, even though the newborn presented no symptoms, this first confirmed case of embryonic mosaicism persisting through gestation to birth emphasizes the need of prenatal testing, particularly amniocentesis, in pregnancies from mosaic embryo transfers.

Recommendations for transfer based on parameters of mosaicism (level, type, chromosome involvement) have been issued before, but those are based on limited experimental data and are therefore largely conceptual [125,171]. A risk scoring system for mosaic embryo transfers has been proposed, basing its rationale on mosaicism and aneuploidies observed in chorionic villus sampling (CVS) data and products of conceptions (POCs) from miscarriages [23]. However, the precise extent to which mosaicism in TE cells reflects placental or fetal mosaicism remains unestablished, and the matched TE biopsy/amniocentesis data mentioned above suggest there is very little correlation. While there is consensus that mosaic embryos have a different set of outcomes than euploids, there is still no agreement among published studies on the specific mosaic features that affect implantation and miscarriage. Conflicting data exist on whether level of mosaicism in the TE biopsy (the percent aneuploid cells) is predictive of outcome [39,40], or whether the type of mosaic (involving segmental, versus whole chromosome, versus complex abnormalities) has an effect [35,37,40]. These reported discrepancies exist even when the same PGT-A platform and guidelines to define mosaicism were used [37,39,40]. The likeliest cause for these inconsistencies is the small sample sizes of the individual studies, making findings of sub-analyses questionable. One report of amassed data from different centers to increase power of analysis has made the following observations evaluating 822 mosaic embryo transfers: There was significant correlation between clinical outcome and level of mosaicism (low level mosaics perform better), as well as type of mosaicism (segmental mosaics perform better than whole chromosome mosaics, and complex mosaics perform worst of all) [168]. This is a rapidly developing topic, and as more clinics become inclined to transfer mosaic embryos in the absence of euploids, expanding data will further clarify these points.

As mentioned above, transfer of mosaic blastocysts should be accompanied by patient counsel with emphasis on prenatal testing (particularly amniocentesis), because data on the exact risk to fetuses are still forthcoming [55,172]. A recent study by Besser and colleagues describing the experience in their center showed that approximately 30% of counselled patients opted for transfer of a mosaic embryo rather than pursuing an additional treatment cycle [173]. The likelihood of undergoing mosaic embryo transfer grew considerably with increasing patient age or number of prior retrievals. Of the patients opting for mosaic embryo transfer, 54.5% pursued amniocentesis.

In summary, the available data indicate that mosaic embryos are viable and can result in seemingly healthy births, albeit with lower success rates than euploids. Ongoing studies will permit further refined ranking of embryos within the mosaic category. The next set of studies should focus on obstetrical and neonatal outcome data from mosaic embryo transfers to obtain a more thorough understanding of their chromosomal and physiological health.

### 6.2. Why Segmental Abnormality Should Be Managed Differently

Recent observations have noted the poor concordance of segmental copy number variations between TE biopsy and ICM (see Section 5.1). The data oblige the field to re-evaluate the category of segmental abnormality, which until now was combined with the conventional aneuploid group and deselected from transfer. This practice likely leads to discarding potentially competent embryos, as a segmental abnormality in a TE biopsy was shown to be predictive of the ICM only ~50% of the time [75,136,137]. Conversely, the transfer of a segmental abnormality embryo should not be performed heedlessly given the fact that ~50% of such embryo profiles do indeed reflect the presence of the same segmental loss or gain in the ICM, and an estimated ~32% of segmental abnormality are meiotic in nature and present throughout the cells of the remaining blastocyst [75]. This would result in failed implantation, miscarriage, or possibly liveborn congenital syndromes if carried to term.

How then should embryos presumed to harbor segmental abnormalities be managed in the clinic? Should some patients treat transfer of an embryo with segmental abnormality as a final alternative, subject to their tolerance for risk and inclination to perform prenatal testing? Interestingly, data indicate that collecting a second TE biopsy of such embryos may facilitate the clinical decision [75,136,137]. Experiments of multifocal biopsies in 31 blastocysts by Navratil and colleagues showed that approximately half of the time a segmental abnormality in the original TE biopsy was discordant with a second TE biopsy and remaining embryo, but in case of concordance between the two TE biopsies, the profile of the remaining embryo reflected the segmental abnormality 94% of the time [137]. In similar experiments analyzing 53 blastocysts, Girardi and colleagues observed that when two TE biopsies were discordant for a segmental abnormality, the likelihood of the ICM containing the segmental abnormality was 21%, but when the two TE biopsies were concordant, the ICM contained the segmental abnormality 84% of the time [75]. Although re-analyzing an embryo in the clinic demands a second freeze-thaw cycle and biopsy as well as another round of PGT-A, it might be the only way to appropriately prioritize embryos within the segmental abnormality category. At least one study has shown that a second round of TE biopsy collection and cryopreservation did not markedly affect implantation outcome or the likelihood of pregnancy complications [118].

Further studies are required in order to evaluate this approach. For now, the data should compel us to differentiate segmental abnormality embryos from whole chromosome aneuploids as two separate categories of PGT-A. A consensus for the clinical management of segmental abnormality embryos is yet to be elaborated.

### 6.3. Refinement of the PGT-A Category System: Is it Necessary?

Expansion of PGT-A categories might admittedly not be an easy change to integrate in medical practice. Clinicians and patients have become accustomed to a binary classification (normal/abnormal). The associated genetic counseling is straightforward, and so is the identification of embryos available for transfer. As a consequence, some have regarded the higher dynamic range and resolution of PGT-A by NGS and its ability to identify mosaicism and segmental abnormality as a nuisance at best and disadvantage at worst.

The strength of the more fragmented classification system lies in better predictability of outcome for each specific embryo. As mentioned above, the observation that blastocysts in the mosaic category have lower implantation potential than euploids has been replicated numerous times [35,37,38,39,40]. To retain the classical binary system means that either mosaic embryos become merged with the normal group, resulting in a lower implantation rate overall, or they become merged with the abnormal group, in which case they will be discarded even though competent. Either option is evidently disadvantageous. Furthermore, if indeed one kind of mosaic blastocyst has higher implantation rates than another, as recent data suggest, it is clearly advantageous to prioritize transfer of the former when the choice is presented. If no other embryos are available, embryos with segmental abnormality could undergo a second round of biopsy collection and PGT-A to identify those with increased potential to result in a healthy birth. This could provide a new opportunity to patients that would otherwise need to resort to further treatment cycles.

Hence, expanding the PGT-A categorization system seeks to: (1) increase overall rates of positive clinical outcome, and (2) identify potentially competent embryos that otherwise would have been deselected. Such clinical benefits should clearly outweigh the increased complexity in the system and associated burden of genetic counseling.

## 7. Current Developments and Future Directions of PGT for Chromosomal Abnormalities

### 7.1. Mitochondrial DNA Quantitation During PGT-A: Where Are We Now

The human genome in its entirety comprises autosomes, sex chromosomes, and the chromosome contained in mitochondria. The mitochondrial chromosome is a 16.5 kb circular double stranded multicopy DNA molecule encoding numerous essential genes for mitochondrial function. Each mitochondrion contains multiple replicas of mitochondrial DNA (mtDNA), and each cell contains numerous mitochondrial organelles. Since those numbers are not static and can fluctuate in response to energetic demands of the host tissue, there is substantial variation in the copy number of mtDNA present in each cell of the human body.

Two independent studies first raised the possibility that quantitation of mtDNA could serve as a biomarker of embryo viability [174,175]. They indicated that blastocysts with PGT-A that failed to implant tended on average to have higher mtDNA content in the TE biopsy, compared to successfully implanted blastocysts. In addition, the studies described a threshold of mtDNA copy number, that always led to failed implantation if it was surpassed.

Since then, the field of mtDNA quantitation in embryology has been turbulent and equivocal. Some studies have supported the initial reports [176,177,178], but many others have refuted them [179,180,181,182,183], showing no predictive power of mtDNA quantitation in TE biopsies regarding clinical outcome. An interesting observation to come out of this debate is that some clinics produce no embryos with substantially elevated mtDNA levels (about half of the 37 participating clinics in one study), whereas some clinics generate upwards of 20% of blastocysts with very high mtDNA copy number [176]. The underlying biological explanation remains to be confirmed, but is very likely tied to embryo staging and morphology. Several studies have reported an inverse correlation between mtDNA levels and embryo cellularity/developmental progression/expansion [179,180,184]. This is reasonable given that no mtDNA replication occurs between zygote and blastocyst stage, and the initial pool of mtDNA molecules becomes diluted between dividing cells [185,186,187]. Therefore, embryos that develop slower will retain more mtDNA copies per cell. If mtDNA copy number quantitation only reflects the embryo’s developmental stage, it is an impractical tool when many clinics perform morphological assessment of embryos and eliminate poorly developing samples from consideration for transfer. Hence, the precise role of mtDNA quantitation in the fertility clinic is still being evaluated, but its routine and universal use to deselect embryos for transfer remains unsubstantiated, particularly after the recent report that blastocysts with disproportionally high mtDNA copy number can result in healthy births [181].

### 7.2. Variations and Add-Ons to Conventional PGT-A

A snapshot of today’s PGT-A landscape reveals WGA-based low-pass/shallow NGS as the workhorse of the industry. The technique has found a balance between three key factors: cost, ease of use, and quality of information generated. Nonetheless, numerous alterations to the standard PGT-A platform have recently emerged or are currently in development.

One is the aforementioned targeted amplification-sequencing method [104], which increases sequencing depth in specific locations of the genome, but usually at the expense of breadth of genome coverage. Recent versions of this technique have taken advantage of repetitive sequences that are spread across the genome, permitting amplification using few primer pairs [188]. Some families of repetitive elements contain appreciable sequence variation, meaning that using a single primer pair in the reaction (reagent savings, added simplicity) potentially provides widely distributed mapping of reads across the genome after bioinformatic analysis. The depth of sequencing is adequate to perform SNP analysis, in addition to quantitation. Allelic ratio analysis can improve confidence of aneuploidy calling, and can provide information regarding haploidy, triploidy, and UPD [189].

There is substantial interest in developing methods to harness the sequence information gathered during NGS, aside from the usual copy number information. For example, Voet, Vermeesch and colleagues have developed haplarithmisis, a method that quantifies sequencing reads to perform PGT-A, but additionally uses the SNP information contained in the sequencing reads to determine haplotypes [190,191]. This can be used to identify embryos with haplotypes carrying disease alleles, but it also reinforces confidence in calling of copy number changes, and reveals the parental and mechanistic origin of chromosomal abnormalities.

Several efforts are underway to combine PGT for chromosomal abnormalities and monogenic conditions (PGT-A/-M) in a single reaction. For example, Zimmerman and colleagues have described a qPCR method to perform simultaneous aneuploidy analysis and single gene testing [192]. Farmer and colleagues have shown that combining a WGA reaction and specific primers targeting the 23 most common mutations in *CFTR* do not compromise NGS analysis of chromosomal abnormalities, and can simultaneously provide accurate single nucleotide variant (SNV) and small indel information for the *CFTR* gene [193]. Such a technique can be adapted to any mutation using appropriate primer spike-ins. Del Rey and colleagues have successfully combined NGS copy number analysis with large panels of common single gene conditions [194]. Alcaraz and colleagues reported a technique that combines chromosomal copy number calling with a SNP-based analysis of a mutation specific to a patient’s genetic profile, showing its feasibility in over 150 different mutations [195].

Handyside and colleagues have developed a technique called karyomapping [196]. It relies on SNP arrays, which aside from providing copy number data, also harness the information contained in near 300,000 SNPs distributed in the genome, permitting linkage analysis for virtually any region of interest. Testing of the subject’s relatives reveals the segregation patterns of mutated loci and flanking informative SNPs, which subsequently provide the framework for linkage analysis in tested embryos. The universal applicability of the technology to virtually any single gene mutation is potentially very advantageous to classical analysis of monogenic conditions in embryos, where each mutation requires a specific assay. In addition to combining PGT-A and -M [197,198], karyomapping allows detection of haploidy and triploidy, as well as UPD.

A recent array-based technology developed by Treff and colleagues combines copy number and SNP analysis, and can simultaneously perform PGT-A, -SR, -M, as well as testing for polygenic (-P) conditions [199,200]. A polygenic risk score (PRS) is calculated for a panel of diseases, estimating the relative likelihood of an embryo to develop conditions such as diabetes or heart disease.

In an effort to improve ease-of-use and lower costs, Wei and colleagues have recently adapted nanopore technology in a MinION instrument (Oxford Nanopore) [201] to PGT-A [202]. While thorough validation is still pending, pilot experiments have shown it can detect aneuploidy and, to some degree, mosaicism in TE biopsies. Compared to standard NGS methods, this technology is estimated to increase sequencing speed by 15,000-fold, has a 99X lower capital equipment cost (~USD 1000), and an instrument footprint the size of a deck of cards.

The limited amount of genetic material in a TE biopsy renders ‘true’ whole genome sequencing a formidable task. However, there have been valiant efforts at obtaining a much broader genome coverage than with standard approaches, typically by coupling high-level sequencing with bioinformatic methods for linkage analysis with familial genomic data. Kumar and colleagues have described a method to infer the entire genome of an embryo from a TE biopsy without performing deep sequencing, instead using SNP linkage analysis with familial genome information [203]. Two studies by Peters et al. [204] and Murphy et al. [205] have shown that detailed assessment of a TE biopsy by high-pass sequencing and linkage analysis can identify new, potentially disease-causing SNPs, in addition to producing copy number and single gene data (PGT-A/-M).

In summary, add-ons to PGT-A can provide valuable supplementary information and/or decrease costs, but should do so without compromising the very core question PGT-A seeks to answer: Are aneuploidies present in the sample? Many of the above tests still need to produce data that convincingly show their copy number analysis is as powerful as conventional WGA-based PGT-A, especially regarding mosaicism and segmental abnormalities.

### 7.3. The Prospect of Non-Invasive PGT-A

A method of PGT-A that does not require physical isolation of cells from the embryo would revolutionize the field. With the decreasing cost of sequencing, the most expensive component of PGT-A has become biopsy isolation, which requires hi-tech laser equipment and greatly skilled embryologists to perform the microsurgery. These hurdles likely prevent some clinics from adopting a PGT-A program. Even though studies have suggested negligible effect on blastocyst viability when TE biopsy isolation is performed correctly [90,118], there is still risk of poorly executed, harmful, or botched procedures and other more nuanced detrimental effects, such as those associated with prolonged temperature and gas fluctuations during the procedure. Furthermore, some embryos do not get tested because poor morphology precludes TE biopsy collection, and a non-invasive method could increase the pool of assessed embryos.

The concepts of minimally invasive (mi-), or non-invasive (ni-)PGT-A became plausible after discovery of (presumably embryo-derived) DNA in blastocoel fluid (BF) [206], and spent culture medium (SCM) [207]. The ensuing question was whether those fluids contained sufficient good quality DNA to evaluate chromosomal copy number. The option involving BF was explored first.

BF is isolated by ‘blastocentesis’ [208], in which a fine needle is inserted through the zona pellucida and between TE cell junctions into the blastocoel cavity, and fluid (< 1 µL) is aspirated. This collapses the blastocyst, which can subsequently be vitrified or left to re-expand. Gianaroli and colleagues have made an extensive exploration on the subject of concordance between BF and embryonic cells, publishing three studies based on WGA and aCGH technology [208,209,210]. The latest, published in 2018, is their largest analysis (*n* = 256 embryos), reporting 94% ploidy concordance between BF and a TE biopsy, and 66% full karyotype concordance [209]. Nevertheless, BF amplification failed in 29% of cases, and of those that amplified a further 13% did not produce an informative chromosome copy number result. An interesting observation was that euploid embryos were significantly more likely to fail BF amplification, possibly pointing at a biological mechanism. The authors hypothesized that DNA in BF is likely to originate from cell apoptosis, which is more frequent in aneuploidy [209]. Therefore, the quantity of DNA in BF might have some prognostic value regarding blastocyst competence.

Studies from other groups, one based on WGA and aCGH [211] and two based on WGA and NGS [127,212], have reported lower concordance of ploidy between BF and TE/ICM biopsy, between 62%–75%, as well as decreased full karyotype concordance, between 38%–48%. Crucially, failed amplification rates for BF showed a large range, between 13%–65%, possibly reflecting different approaches to blastocentesis, BF storage, and amplification chemistry [127,211,212], but also differences in blastocyst population analyzed and true incidence of euploidy, mosaicism, and aneuploidy.

Several groups have recently explored the alternative method (which would be truly non-invasive): analysis of SCM. DNA contamination has been an obvious concern, since some media formulations contain foreign DNA, and there is potential for carryover maternal DNA in the culture drop. However, the observation that the total amount of DNA increases with developmental progression [213,214] would indicate that the bulk DNA derives from the embryo. The first notable SCM study was performed by Shamonki and colleagues in 2016, reporting that their system (Qiagen’s Repli-G kit for amplification and aCGH) produced suboptimal data for 96% of the 56 samples analyzed [215]. This confirmed that chemistries designed for chromosomal evaluation from cellular biopsy would unlikely be transferable to niPGT-A in their current formats. A series of studies followed, testing different methods of DNA amplification (MALBAC or SurePlex/PicoPlex, with various modifications), analysis (aCGH or NGS), and culturing protocols, expertly detailed by Leaver and Wells [216].

The biggest caveat in many of the publications to date is that embryos were subjected to some form of manipulation before SCM was collected [217,218,219,220,221]. Putting embryos through Day 3/4 assisted hatching, a freeze-thaw cycle, and/or a cellular biopsy prior to SCM collection provides a source of added DNA in the medium, confounding the subsequent analysis of amplification rate and concordance to the embryo. While it should not entirely invalidate such studies, these shortcomings must be carefully considered.

For the most part, studies with no embryo manipulation prior to SCM collection will be discussed here, as they can give a true sense of whether niPGT-A is applicable in the clinic. Liu and colleagues evaluated 88 SCM samples after continuous culture between Days 1–5, using MALBAC amplification (Yikon Genomics) and NGS on a HiSeq 2500 (Illumina) instrument, reporting a 91% amplification rate [222]. Ploidy concordance with a TE biopsy was observed in 84% of cases (although mosaic and aneuploid results were conflated into one ‘abnormal’ group), and full karyotype concordance was 65%. Only later studies revealed that maternal DNA contamination from the oocyte retrieval procedure is a significant concern, and the uninterrupted culture from zygote to blastocyst stage likely maximizes that problem. The same testing method, namely MALBAC amplification (Yikon Genomics) and NGS on a HiSeq 2500 instrument (Illumina), was used in a study by Fang and colleagues, this time washing embryos at Day 3 of culture and moving to new media drops until Day 5 or 6 [223]. The study reports a 97% amplification rate in the 170 samples analyzed. The authors did not collect a TE biopsy to perform concordance analysis, and instead embryos were transferred in the clinic, selecting them according to the result of the test (which they call ‘NICS’, for noninvasive chromosome screening). In total, 52 blastocysts that had been classified as normal were transferred to 50 patients, resulting in 30 implantations (58% rate), 3 miscarriages (10% rate), and 27 births (52% rate per embryo transfer). Those results could be regarded as favorable rates of positive clinical outcome, but unfortunately the study did not have a control arm, making the results difficult to evaluate. Are those outcomes better than if no niPGT-A had been performed? And would TE biopsy PGT-A have resulted in better outcomes? Two ongoing trials, in which NICS is compared to morphology assessment alone (ClinicalTrials.gov ID: NCT04339166) or to conventional PGT-A (ID: NCT03879265) will shed light on these questions.

A study by Vera-Rodriguez and colleagues [214] explored three important issues concerning niPGT-A: (1) Amount of DNA, (2) Maternal DNA contamination, and (3) Embryonic mosaicism. To explore those concepts, the authors used a high-performance DNA quantitation method, SNP analysis to discern maternal from embryonic DNA, and FISH to evaluate each cell in a subset of blastocysts. The authors analyzed 56 SCM samples collected at Day 5 of culture (after having performed a media change at Day 3). Detailed DNA quantitation showed a median 6.7 pg DNA, which is approximately equivalent to the DNA content of one diploid cell- with half of the samples containing fewer amounts. In comparison, no-embryo control drops contained a median 1.4 pg. It must be stressed that this particular study did perform assisted hatching at Day 3, meaning that a truly non-invasive protocol might result in lower amounts of DNA per SCM. There was no difference in the amount of DNA between euploid and aneuploid embryos, in contrast to what was observed in BF studies [209]. Strikingly, SNP analysis revealed maternal DNA presence in all samples tested, on average constituting a remarkable 92% of all DNA in SCM, even though a media change had been performed at Day 3. To explore the ploidy of SCM, the authors employed a double WGA amplification technique of SurePlex WGA (Illumina), followed by a complete IonReproseq (ThermoFisher) protocol and sequencing on an Ion PGM instrument (ThermoFisher). Ninety-one percent of samples yielded a result, compared to 100% using TE biopsies. FISH analysis of complete blastocyst cellular makeup revealed that SCM samples poorly reflected the status of embryos that were mosaics, and TE biopsy mosaicism was a far superior predictor of mosaicism in the remaining blastocyst. This was attributed to the possibility that in a mosaic context, cells with different ploidy release their DNA content into the SCM at different rates- rendering the SCM a poor representative of the embryo. Together, these findings clearly identified some of the hurdles that need to be addressed in developing niPGT-A for clinical use.

In 2019 the same group published a prospective blinded study, in which Rubio and colleagues analyzed 115 samples and showed a 95% amplification rate with 79% overall ploidy concordance to a TE biopsy and 64% full karyotype concordance (including segmental abnormalities) [224]. To perform niPGT-A, the group used a modified version of the IonReproseq protocol (ThermoFisher), followed by sequencing on an Ion S5TM XL system (ThermoFisher). Embryo culture did not involve assisted hatching, and embryos were thoroughly washed at Day 4 through three media drops in hopes of removing carryover maternal DNA and lingering cumulus cells. Each embryo was subsequently placed in a new reduced media drop of 10 µL (to ensure the entire sample could be included in a WGA reaction) and cultured to Day 5, 6, or 7, at which time SCM was collected. Sub-analysis showed that extended culture to Day 6 or 7 (≥48 h) improved results (compared to just 24 h), increasing the overall ploidy concordance with a TE biopsy to 84% and full karyotype concordance to 72%, as well as rate of amplification to 100%. This considerable enhancement clearly showed that extended time in culture increases the amount of embryo-representative DNA in the SCM. This study went on to show in a small sample group (*n* = 29) that embryos had an improved chance of implantation and lower miscarriage when TE biopsy and SCM concorded on euploidy status, compared to discordant cases for which TE biopsy indicated euploidy but SCM showed aneuploidy. If these results are replicated in a larger sample group, one could envision niPGT-A as an adjunct to conventional PGT-A to increase likelihood of favorable outcome. Two ongoing studies by this group (ClinicalTrials.gov IDs: NCT03520933 and NCT04000152) are further assessing the value of niPGT-A in the clinic.

A different approach altogether, combining BF and SCM, has so far been explored in a few studies that have all reported high rates of amplification 98%–100% [225,226,227]. Perhaps the most compelling are the efforts by Kuznyetsov and colleagues [226], since their protocol excluded freeze/thaw cycles prior to sample collection. In a publication from 2018, the Kuznyetsov group analyzed 19 fresh culture samples from 9 patients. Embryos were placed in new culture drops at Day 4 and left to grow until Day 5 or 6, at which point a TE biopsy was collected and the blastocyst was further collapsed with the use of lasers, allowing the BF to emanate into the SCM. The fact that a TE biopsy was collected prior to collection of fluids is a caveat, since the process likely releases DNA into the medium. An unspecified amount of the 25 µL combined BF and SCM sample was subsequently processed in one reaction by SurePlex amplification (Illumina) and VeriSeq NGS (Illumina) on a MiSeq instrument (Illumina) (the same protocol as the corresponding TE biopsies). The combined BF-SCM samples and corresponding TE biopsies had 100% ploidy concordance, and 100% full karyotype concordance for whole chromosome events (which decreased to 71% if segmental abnormalities and mosaicism were included).

Kuznyetsov and colleagues subsequently published a study in 2020 with a larger sample size (*n* = 145) excluding the prior TE biopsy collection, reporting a 100% amplification rate and a 97.8% concordance (euploid/aneuploid) with TE samples [228]. When mosaicism was considered (observed either in cell-free DNA or TE biopsy), rate of concordance declined. One parameter still required optimization: 88.2% of BF-SCM sample amplifications yielded informative NGS results, compared to 98% of TE biopsy amplifications. Overall, those are promising observations regarding the clinical application of this strategy. Since the protocol included assisted hatching at Day 4, an outstanding question is whether omitting this step (in an effort to further reduce the manipulations/invasiveness of the process) would influence the reaction. Other interesting findings from the study include that blastocyst morphology did not correlate with quantity of cell-free DNA, and the cell lysis step could be omitted from the WGA reaction (as it might contribute to maternal DNA contamination though carryover cumulus/corona cells).

There have also been efforts to quantify mtDNA levels in SCM samples. Stigliani and colleagues have reported that higher levels of cell-free mtDNA might be predictive of implantation in cleavage day embryo transfers [206]. Compared to embryos that failed to implant, they observed a threefold increase in mtDNA quantity (on average) in embryos that implanted, but these findings are yet to be replicated with a larger sample size (*n* = 51 not implanted, *n* = 43 implanted embryos, *p* = 0.0452). The same group also observed that higher quantities of cell-free mtDNA at Day 3 of culture have predictive power regarding the likelihood of reaching the blastocyst stage [229]. Whether cell free mtDNA quantitation may serve as a biomarker of implantation for blastocyst stage transfers remains to be explored.

Valuable lessons have been learned from the studies so far: (1) Manipulation of embryos prior to sample collection (freeze-thaw cycle, biopsy collection, assisted hatching) might release DNA into media, compromising a study’s results regarding truly non-invasive PGT-A. Those manipulations should be avoided in future studies, (2) Fully euploid and aneuploid embryos might release similar amounts of DNA into the medium, but in a mosaic context, aneuploid cells might preferentially shed their content, adulterating the representativeness of the SCM in regards to the embryo, (3) The minute amounts of DNA require new adaptations of PGT-A chemistries and protocols that accommodate as much of the culture drop volume as possible, (4) Maternal DNA contamination is a big concern, (5) Longer cultures to Day 6 or 7 provide more cell-free (cf) DNA.

Still, several questions persist: (1) What is an acceptable rate of false or no results in niPGT-A (considering its many benefits)? (2) What is the biological mechanism that releases embryonic DNA into surrounding fluids? (3) Are SCM and/or BF better or worse representatives of the true ploidy status of the blastocyst, compared to a TE biopsy? (4) What is the capacity of niPGT to detect segmental abnormalities and mosaicism?

Together, the SCM and BF studies have revealed that the naturally small amount of DNA present in those fluids, possibly coupled with compromised integrity and maternal DNA contamination, present a real challenge in the development of niPGT-A. The reports have evidently pushed against the lower limits of detection of existing PGT-A platforms. There is however, cause for optimism; better cell-free DNA amplification chemistries and optimized bioinformatic analyses will bring us closer to an accurate niPGT-A screen suitable for routine clinical use.

## 8. Concluding Remarks

PGT-A is undergoing steady, global expansion; in 2019, the technology had a presence in at least 45 countries [7]. To illustrate its potential for growth, between 2014 and 2016 its utilization increased from 13% to 27% of all IVF cycles performed in the USA [230]. The methodology is constantly evolving, having undergone several rounds of transformative changes since its inception- even in name from PGS to PGD-A, to its current form, PGT-A. Each step has incorporated new approaches (chemistries, biopsy collection technique, bioinformatic tools) and has harnessed novel biological insights of aneuploidy (mosaicism, segmental abnormalities). Contemporary technologies make it possible to identify chromosomal abnormalities in greater detail than ever before, and clinical data call for an expansion of the PGT-A grouping system to include the categories of mosaicism and segmental abnormalities.

The current enthusiastic pursuit of niPGT-A may bear fruit in the near future. Time will tell whether its many benefits might come at the expense of data quality and genome resolution, or whether technical advances will be able to bridge those gaps. In parallel, numerous ongoing and ambitious efforts are developing ways to retrieve an ever-increasing amount of information from a single biopsy in order to achieve a more complete genomic profile and enhanced prognostic assessment of the embryo.

PGT-A is inherently limited because it does not (and probably never will) reflect the chromosomal state of the entire embryo with 100% accuracy, and cannot perfectly predict an embryo’s clinical outcome. However, in many settings, its demonstrated capability to improve likelihood of positive outcome is undeniable and tremendously valuable. Thousands of past, current, and future infertility patients would certainly agree.
